# Potential Value of Visfatin, Omentin-1, Nesfatin-1 and Apelin in Renal Cell Carcinoma (RCC): A Systematic Review and Meta-Analysis

**DOI:** 10.3390/diagnostics12123069

**Published:** 2022-12-06

**Authors:** Sugania Malar Chinapayan, Shanggar Kuppusamy, Ning Yi Yap, Komathi Perumal, Glenda Gobe, Retnagowri Rajandram

**Affiliations:** 1 Department of Surgery, Faculty of Medicine, Universiti Malaya, Kuala Lumpur 50603, Malaysia; 2 Laboratory, Subang Jaya Medical Centre, Subang Jaya 47500, Malaysia; 3 epink Health Sdn Bhd, Shah Alam 40150, Malaysia; 4 NHMRC Chronic Kidney Disease Centre of Research Excellence, The University of Queensland, Brisbane 4067, Australia; 5 Faculty of Medicine, The University of Queensland, Brisbane 4067, Australia; 6 Kidney Disease Research Collaborative, Translational Research Institute, Princess Alexandra Hospital, The University of Queensland, Brisbane 4102, Australia

**Keywords:** adipokine, kidney cancer, marker, prognosis, obesity

## Abstract

Renal cell carcinoma (RCC) is the most lethal genitourinary malignancy. Obesity is a risk factor for RCC development. The role of adipokines in the relationship between obesity and RCC requires confirmatory evidence in the form of a systematic review and meta-analysis, specifically for visfatin, omentin-1, nesfatin-1 and apelin. A search of databases up to July 2022 (PubMed, Web of Science and Scopus) for studies reporting the association of these selected adipokines with RCC was conducted. A total of 13 studies fulfilled the selection criteria. Only visfatin (*p* < 0.05) and nesfatin-1 (*p* < 0.05) had a significant association with RCC. Meanwhile, apelin and omentin-1 showed no association with RCC. The meta-analysis results of nesfatin-1 showed no association with early-stage (OR = 0.09, 95% CI = −0.12–0.29, *p* = 0.41), late-stage (OR = 0.36, 95% CI = 0.07–1.89, *p* = 0.23) and low-grade (OR = 1.75, 95% CI = 0.37–8.27, *p* = 0.48) RCC. However, nesfatin-1 showed an association with a high grade of the disease (OR = 0.29, 95% CI = 0.13–0.61, *p* = 0.001) and poorer overall survival (OS) (HR = 3.86, 95% CI = 2.18–6.85; *p* < 0.01). Apelin showed no association with the risk of RCC development (mean difference = 21.15, 95% CI = −23.69–65.99, *p* = 0.36) and OS (HR = 1.04, 95% Cl = 0.45–2.41; *p* = 0.92). Although the number of studies evaluated was limited, analysis from this systematic review and meta-analysis indicate that visfatin and nesfatin-1 were elevated. In summary, these adipokines may play a role in the development and progression of RCC and hence may have potential diagnostic and prognostic capabilities for RCC.

## 1. Background

Renal cell carcinoma (RCC) is one of the most prevalent urological malignancies, and over 90% of malignant kidney tumours are clear cell RCCs (ccRCC) [1,2]. Globally, cancer incidence has been slowly increasing during the past decade. There is increasing evidence that RCC has a multifactorial aetiology. There are several major risk factors contributing to RCC development, such as smoking [3], consumption of alcohol [4], obesity [5], hypertension [6], reproductive and hormonal factors [7], lack of physical activity [8], diet [9], occupation [10], the environment [11], and genetics and pre-existing comorbidities [12].

Although obesity is recognised as one of the major risk factors in the pathogenesis of RCC, the obesity–RCC association has not been carefully studied and so represents a gap in the literature. The previous literature has linked RCC and obesity to peptide hormones called adipokines, secreted by adipocytes [13,14,15]. Adipokines function to regulate physiological processes that play an overall role in appetite and energy balance, such as lipid metabolism, glucose homeostasis, insulin sensitivity, angiogenesis, blood pressure and inflammatory processes [16]. During obesity, enhanced secretion of adipokines are major factors in cell proliferation, increased cell migration and, subsequently, cancer metastasis and carcinogenesis [16]. The link between obesity, metabolic imbalance, and RCC has given rise to studies on the role of adipokines in the development and progression of RCC. Of the few adipokines that have been explored in RCC, leptin/leptin receptor [17] and adiponectin [18] have been the most studied. A lower circulating adiponectin concentration is associated with an increased risk of RCC. Adipokines induce proliferation and apoptosis in RCC, by up-regulating the p-AMPK and Bcl-xL levels [19]. Leptin was shown to promote cell migration of RCC cells via the activation of the PCP/JNK signalling pathway [20]. In contrast, leptin had contradictory findings: its expression was not associated with the development and prognosis of RCC [21]. Other adipokines, such as visfatin, omentin-1, nesfatin-1 and apelin, have also been linked to cancer development [22]. However, the mechanism by which these adipokines may contribute to the development and progression of RCC is still unclear. 

It is crucial to determine the clear role of these adipokines in RCC development and progression and their potential as prognostic or predictive factors for improving patient management. Thus, in this paper, we explore the potential role and expression of visfatin, omentin-1, nesfatin-1 and apelin in the development and progression of RCC and their ability as biomarkers in RCC.

## 2. Method

### 2.1. Search Strategy

A systematic review was conducted according to the protocol registered in the International Prospective Register of Systematic Reviews (PROSPERO), under identification number CRD42022363925, respecting the recommendations of the Cochrane Collaboration reference for the preparation and publication of systematic reviews and meta-analyses. The results are presented according to the PRISMA guidelines [23]. The following databases were systematically searched for relevant studies published from 2000 to July 2022: PubMed, Web of Science and Scopus. A comprehensive MeSH and text word search terms were developed using the following combination of keywords: ‘Cancer’ or ‘Malignancy’ or ‘Neoplasm’ or ‘Carcinoma’ or ‘Tumour’ or ‘Survival’ or ‘ Disease progression’ or ‘Metastatic’ or ‘Proliferation’ or ‘Angiogenesis’ or ‘Metabolomics’ or ‘Proteomics’ or ‘Diagnosis’ or ‘Gene studies’ or’ ‘Cell death’ or ‘apoptosis’ or ‘Stage’ or ‘Grade’ were combined with ‘Apelin’ or ‘APLN’ or ‘APLNR’ or ‘apelin receptor’ or angiotensin-like-receptor 1 or ‘Omentin’ or ‘Omentin Rs2274907 or ‘Intelectin-1′ or ‘Visfatin’ or ‘nicotinamide Phosphoribosyltransferase’ or ‘NAMPRTase’ or ‘NAMPT’ or ‘PRE-B-CELL COLONY ENHANCING FACTOR-1’ or ‘PBEF-1’ or ‘Nesfatin-1’ or ‘NUCB2’ or ‘Nucleobindin 2’ or ‘Nesfatin-1’. The search terms were modified for each database. 

### 2.2. Inclusion and Exclusion Criteria

**Figure 1:**
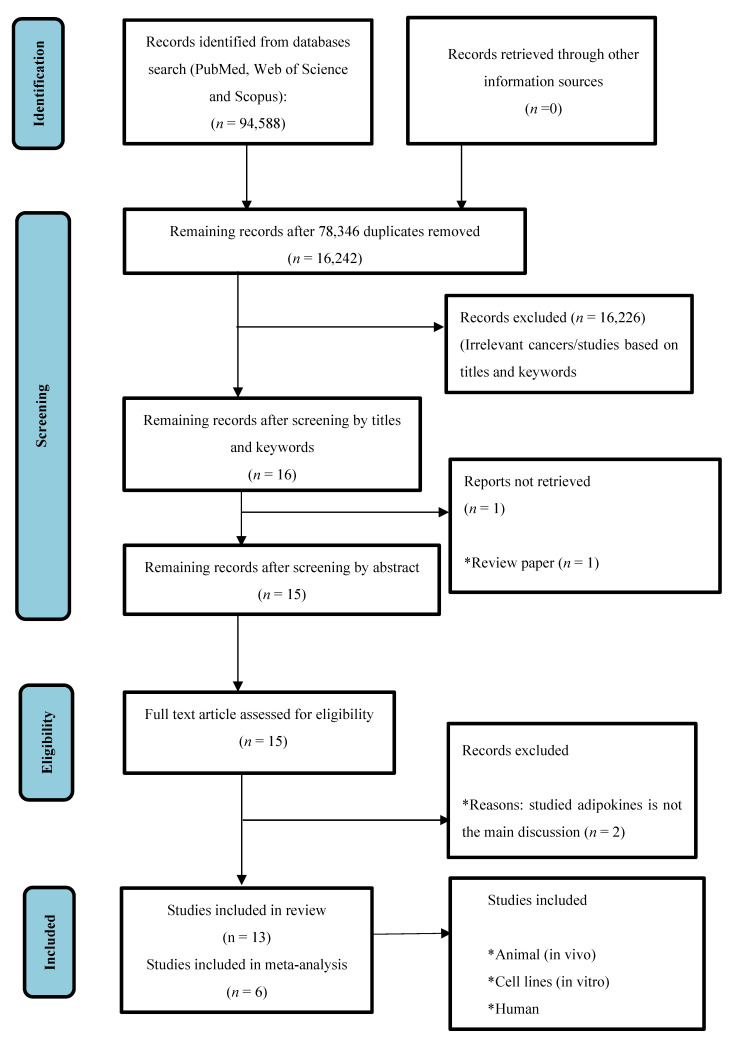
The PRISMA diagram. PRISMA diagram shows the selection process of the studies included in the meta-analysis. * shows reasons that studies were included or excluded.

In total, two reviewers (SMC and NYY) independently evaluated the retrieved studies based on the titles, abstracts and contents. Studies that were included in the analysis met the following criteria: (1) study of RCC and the expression of visfatin, omentin-1, nesfatin-1 or apelin detected in patients’ tissue/serum, RCC cell line or RCC animal model; (2) visfatin, omentin-1, nesfatin-1 or apelin expression was used to assess survival or clinicopathological parameters; (3) original full-text articles published between 2000 and 2022; and (4) studies published in English only. The meta-analysis was performed using articles with sufficient data such as number of patients and controls, stage and grade of RCC, prognostic outcomes and/or diagnostic results, hazard ratio (HR), medians and ranges, means and standard deviations. Abstracts, review articles, letters to the editor or comments, and duplicates from the various searches were all excluded in this review. The PRISMA flow diagram was generated to outline the steps taken for study inclusion or exclusion (Figure 1).

### 2.3. Extraction of Data for Hypothesis Evaluation

All pertinent data were independently extracted by two investigators (SMC and NYY) into a case report form in an Excel spread sheet format. The main data extracted from these articles included: first author, country, year of publication, sample size (number of patients), sample type (cell line/animal/human) and assay/technique. The determination of the diagnostic and prognostic value of the selected adipokines was performed using the following data: (1) number of patients and controls; (2) sample type; (3) stage and grade of RCC; (4) prognostic outcomes and/or (5) diagnostic results. The HR and 95% CI were extracted for the univariate survival outcome of the high adipokine expression group. For studies reporting outcomes as medians and ranges, the means and standard deviations were calculated using the methods described in the previous literature [24].

### 2.4. Assessment of Study Quality

The quality of included articles was independently assessed by two authors (SMC and NYY) using criteria formulated in the Reporting Recommendations for Tumour Marker Prognostic Studies (REMARK) guidelines for immunohistochemistry (IHC)/gene studies [25] and Animal Research: Reporting of In Vivo Experiments (ARRIVE) guidelines for reporting in vivo experiments in animal research [26] and quality assessment tools for in vitro studies adapted from the operationalised Nature reporting checklist. https://osf.io/zr6sc/ accessed on 20 December 2021.

### 2.5. Data Analysis

Statistical analyses were performed using RevMan version 5.4 for Windows (The Cochrane Collaboration). For prognostic values, RCC stages and grades were grouped into early and late stages based on clinical and pathological findings in the literature. The methods outlined in the Cochrane Handbook for Systematic Review of Interventions were used to create the grouping. The data for different outcomes were pooled in a meta-analysis by using RevMan version 5.4. The significance of the examined adipokines for each outcome was pooled as mean difference, odds ratio and hazard ratio, respectively. Survival data such as overall survival (OS) were expressed as the HR and its 95% confidence interval (CI), and differences were considered statistically significant at *p* < 0.05. Statistical heterogeneity among the included studies was analysed using the chi-square test, while the I2 test was used for quantitative analysis of heterogeneity. *p* values less than 0.1 and/or I2 greater than 50% were considered to be high degrees of between-study heterogeneity, in which case a random effects model was used; otherwise, a fixed effects model was used. All studies, including those not suitable for meta-analyses, were subjected to qualitative analysis. For qualitative analysis, study characteristics, main outcomes on expression of studied adipokines and other possibly relevant outcomes were extracted.

## 3. Results

### 3.1. Study Selection and Study Characteristics

A comprehensive literature search from the three databases yielded a total of 94,588 publications. After the removal of duplicates, and non-relevant studies based on the keywords, titles, abstracts and detailed evaluation, 15 full-text articles were inspected for inclusion in this review. A further two articles were excluded, as these two articles did not investigate and discuss the adipokines of interest as their main theme in the papers. Therefore, 13 studies fitting the inclusion criteria were included in the review: visfatin (*n* = four), omentin (*n* = one), nesfatin-1 (*n* = five) and apelin (*n* = four) (Table 1). Out of 13 studies, meta-analysis was only performed for nesfatin-1 (*n* = two) and apelin (*n* = four), as we found insufficient comparative data for visfatin and omentin-1.

### 3.2. Quality of Studies

The quality of each study for human in vivo and in vitro experiments for the evaluation of adipokines in RCC is shown in Supplementary File S1. In these tables, studies with REMARK and ARRIVE scores of more than 10 were considered of good quality.

### 3.3. Summary of Systematic Review Findings 

#### 3.3.1. Visfatin

Overall, four studies investigated the association of visfatin with RCC patients for diagnostic value, prognostic value (stage/grade) and OS. Literature was not available for in vivo and in vitro studies. Visfatin levels were found to be higher in RCC tissue and plasma compared to adjacent normal and healthy controls, respectively [27,29]. There was a positive correlation between the plasma level of visfatin and the T stage of RCC [29]. The high expression of visfatin in relation to higher Fuhrman grades has also been shown in RCC [30]. One study revealed that RCC patients with a high expression of the visfatin gene have a poorer prognosis [28] (Table 2).

#### 3.3.2. Omentin-1

Only one study was found investigating the association of omentin-1 in the serum of RCC patients for diagnostic and prognostic values (stage/grade). The expression of omentin-1 in RCC patients was lower compared to healthy individuals, and there was no significant association between circulating omentin-1 levels with any stage of RCC [31] (Table 2).

#### 3.3.3. Nesfatin-1 

A total of five studies were found investigating the association of nesfatin-1 with prognosis and OS as well as functional investigations. There is a lack of literature identifying the diagnostic value of nesfatin-1. The high expression of nesfatin-1 in relation to higher stages and Fuhrman grades indicated the association with poorer prognosis in RCC [32,33,34]. In vitro studies showed RCC cell lines had a high expression of nesfatin-1. Knock-down experiments of nesfatin-1 prevents RCC invasion by facilitating apoptosis [35,36]. Furthermore, the suppression of nesfatin-1 also inhibited tumour nodule formation in a murine RCC tumour model (Table 3).

#### 3.3.4. Apelin 

A total of four studies investigating the association of apelin in RCC patients were found for the diagnostic value and prognostic value (stage/grade), as well as for OS. There has not been any in vivo and in vitro studies performed for apelin. The results were shown to have contradictory diagnostic values among studies. One study based on an online database, such as the gene expression profile of GSE6344, showed the gene expression of apelin is high in RCC tissue compared to normal adjacent tissue [27]. The same study also illustrated an insignificant difference in mRNA expression between RCC and adjacent normal tissue [27]. Another study showed apelin was highly expressed in RCC tissue compared to normal tissue [39]. Other studies have shown a very weak association with the tumour histological grade of RCC, and a high expression of this protein significantly correlated with overall survival in RCC patients [37,38].

### 3.4. Meta-Analysis Outcomes 

#### 3.4.1. Nesfatin-1

**Figure 2:**
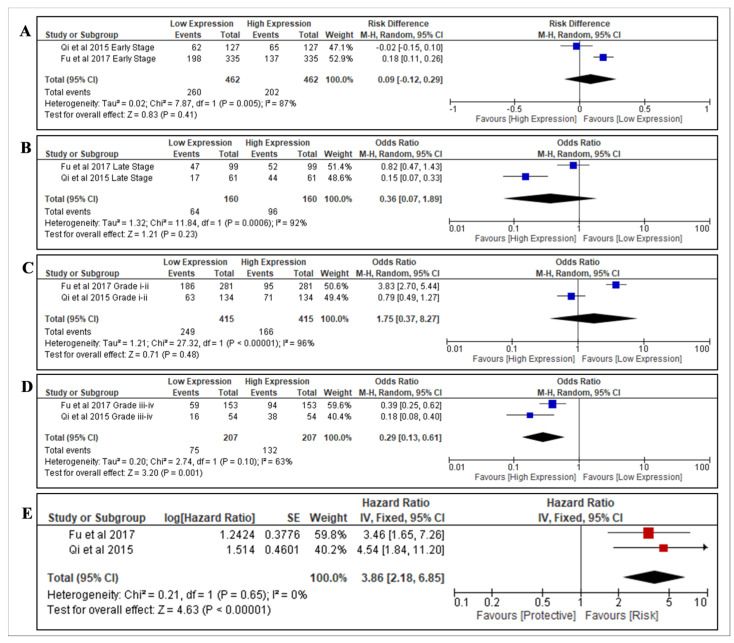
Forest plots of included studies on prognostic value of nesfatin-1. (**A**) Prognostic value of nesfatin-1 in RCC localised disease (Early Stage). (**B**) Prognostic value of nesfatin-1 in RCC advanced disease (Late Stage). (**C**) Prognostic value of nesfatin-1 in localised RCC disease (Low Grade). (**D**) Prognostic value of nesfatin-1 in advanced RCC disease (high grade). (**E**) Expression of nesfatin-1 on the OS in RCC. Odds ratios, hazard ratios and 95% confidence intervals (95% CIs) were pooled using random effects and fixed effects meta-analysis. The squares indicate the effect size for each study (ORs between localised disease and advanced disease groups and HR of overall survival of nesfatin-1) and the length of the lines indicates the 95% CIs. The size of the square represents its weight in the analysis. The black diamond on the bottom of the forest plot indicates the overall weighted effect size. I^2^ indicates between-study heterogeneity. Abbreviations: M-H = Mantel–Haenszel; CI = confidence interval [32,33].

Overall, two studies with sufficient data were included in the meta-analysis to determine the association of nesfatin-1 with the stage, grade and OS for RCC. The prognostic value (stage/grade) and OS of RCC tissue based on localised (*n* = 462) and advanced tumour stages (*n* = 160) were evaluated [32,33]. This evaluation was also performed for low-grade (*n* = 415) and high-grade tumours (*n* = 207) [32,33]. In the overall analysis, there was no significant association in the expression of nesfatin-1 in localised early-stage (OR = 0.09, 95% CI = −0.12–0.29, *p* = 0.41) and late-stage (OR = 0.36, 95% CI = 0.07–1.89, *p* = 0.23) RCC, respectively (Figure 2A,B). There was also no association found with low-grade RCC (OR = 1.75, 95% CI = 0.37–8.27, *p* = 0.48) (Figure 2C). However, nesfatin-1 expression was significantly associated with high-grade RCC (OR = 0.29, 95% CI = 0.13–0.61, *p* = 0.001) (Figure 2D) and significantly correlated with poorer OS in RCC (HR = 3.86, 95% Cl = 2.18–6.85; *p* < 0.01) (Figure 2E). This indicates that nesfatin-1 may be a predictor for aggressive RCC.

#### 3.4.2. Apelin

**Figure 3:**
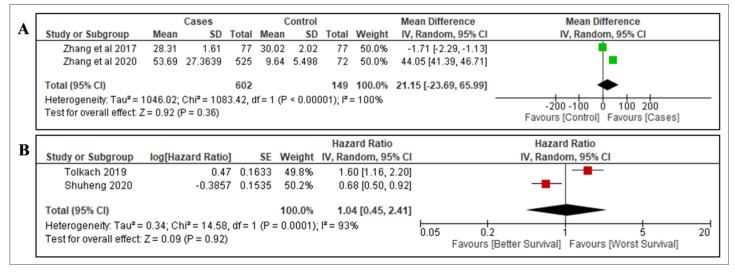
Forest plots of included studies on expression of apelin in RCC. (**A**) Expression of apelin in RCC versus adjacent normal tissue as a control [27,39]. (**B**) Apelin expression and OS in RCC. Mean difference, hazard ratio and 95% confidence intervals (95% CIs) were pooled using random effects meta-analysis. The squares indicate the effect size for each study (mean difference between control and RCC cases of apelin and HR of overall survival of apelin) and the length of the lines indicates the 95% CIs. The size of the square represents its weight in the analysis. The black diamond on the bottom of the forest plot indicates the overall weighted effect size. Abbreviations: I^2^ = between-study heterogeneity; CI = confidence interval [37,38].

Overall, four studies were used to analyse the association of apelin with the diagnostic value and OS of RCC. There was insufficient data for analysis of the prognostic value in terms of stage and grade. Zhang et al. (2017) and Zhang et al. (2020) evaluated the gene expression of apelin in RCC (*n* = 602) compared to adjacent non-cancerous tissue as a control (*n* = 149) [27,39]. There was no significant association between the gene expression of apelin and RCC (mean difference = 21.15, 95% CI = −23.69–65.99, *p* = 0.36) (Figure 3A). We found another two studies that evaluated the gene expression levels of apelin in RCC tissue with regards to OS [37,38]. The expression of apelin did not correlate significantly with OS in RCC (HR = 1.04, 95% Cl = 0.45–2.41; *p* = 0.92) (Figure 3B).

## 4. Discussion

Adipokines, collectively referred to as hormones and cytokines derived from adipose tissues, have been recognised as one of the main pathological characteristics of obesity [40]. There has also been an increasing recognition of their roles in the initiation, progression and metastasis of various tumours, including RCC [41]. The emerging adipokines in relation to RCC explored in this paper are visfatin, omentin-1, nesfatin-1 and apelin. Our study presents a systematic review and meta-analysis of relevant adipokines in RCC in relation to the diagnostic and prognostic values for stage, grade and OS.

The first adipokine reviewed was visfatin and its expression in RCC. Visfatin is known for cellular homeostasis of nicotinamide adenine dinucleotide (NAD), which regulates cell viability, including the survival of cancer cells [42]. As per our review, visfatin showed a significant correlation with the development and prognosis of RCC. Visfatin levels were upregulated in RCC compared to normal healthy controls. The levels were significantly related to the T stage of the disease [29]. In addition, high expression levels of the visfatin gene were also associated with a poorer survival outcome. This supports the use of visfatin as a prognostic marker and a potential target for the treatment regimen for this cancer [42]. Visfatin is also under investigation as a potential biomarker in various types of cancers, as it is upregulated in tissue or plasma of patients with cancers such as oral squamous cell carcinoma, pancreatic ductal adenocarcinoma, breast cancer and thyroid malignancy based on a pan-cancer scale [42]. Accumulating evidence suggests that visfatin can function as a growth factor or as a cytokine through several molecular mechanisms, including signalling pathways involving PI3K/Akt, ERK1/2 and STAT3 [16,43]. Several studies have also proposed that visfatin, as an adipocytokine, can exert insulin-like effects, such as stimulating glucose uptake and cell proliferation by activating the insulin downstream pathway. However, its exact oncological role in RCC remains to be determined. Thus, further evidence from functional experiments and larger prospective and longitudinal studies is required. 

The second adipokine reviewed was omentin-1 and its expression in RCC. Omentin-1, also known as intelectin-1, is abundantly expressed in human visceral adipose tissue and inverse associations between circulating omentin-1 and obesity have been demonstrated, while aberrant serum omentin-1 with either increased or decreased levels has been reported in solid malignancies [44]. Patients with RCC had statistically lower circulating omentin-1 levels compared to the healthy control group. In addition, the association with TNM staging for RCC is not significant for the circulating omentin-1 levels. These findings are in accordance with a recent study on the gene expression of omentin-1 and its circulating levels, which found a low concentration in patients with breast cancer compared to healthy controls [45]. Furthermore, it has been postulated that omentin-1 has tumour suppressor gene activity. Zhang et al. (2013) demonstrated that, by decreasing p53 deacetylation, omentin-1 could increase p53 protein levels by activating the sirt1 deacetylase. As a result, the stability of the p53 protein is increased, promoting apoptosis and preventing carcinogenesis [46]. Consistent with the above findings, omentin-1 may have an independent protective role in cancer biology and potentially be a therapeutic target. However, in contrast, there are clinical studies that showed an elevation in serum omentin-1 levels in patients with prostate [47] and colorectal [48] cancers. Due to the above heterogeneous findings among the different cancers and the lack of literature to support the use of omentin-1 as a reliable biomarker, larger multicenter studies are needed to further elucidate the role of omentin-1 in tumourigenesis.

The next adipokine reviewed was nesfatin-1 and its expression in RCC. Nesfatin-1, as a metabolic factor, is a neuropeptide that serves an important role in regulating food intake and energy homeostasis [49]. Nesfatin-1 is expressed in many tissues and performs a variety of physiological functions, such as anti-inflammation, reducing cardiovascular risk and atherosclerosis extent [50]. Recently, nesfatin-1 has also been declared to play a role in the proliferation, invasion, and migration of tumour cells and affects the prognosis of cancer patients [50]. In our review, nesfatin-1 showed a significant correlation with the development and prognosis of RCC patients. The protein expression level was positively correlated with Fuhrman grade and was shown to have poorer prognosis. Our meta-analysis showed that this adipokine was not associated with low-grade stages of RCC; however, it was positively associated with high-grade stages and poorer OS in RCC patients. Therefore, more work is required in this area to verify the diagnostic and prognostic capability of this adipokine. In vivo and in vitro studies revealed the role of nesfatin-1 in cancer progression, particularly in the process of the development and invasion in RCC by increased EMT through the AMPK/TORC1/ZEB1 signalling pathway [35,36]. Studies of female reproductive system malignancy showed contrasting mechanisms, with nesfatin-1 inhibiting proliferation and increasing apoptosis in cell line studies [51]. Hence, it is probable that nesfatin-1 has high tissue specificity and executes its mechanism via varying signalling pathways.

The last adipokine reviewed in our meta-analysis of RCC development and progression was apelin. Apelin is an endogenous peptide, which is expressed in many organs such as the brain, placenta, heart, lungs, kidneys, pancreas, testes, prostate and adipose tissues [52]. Apelin expression is increased in various kinds of cancer and the apelin/APLNR axis plays a key role in the development of tumours through enhancing angiogenesis, metastasis, cell proliferation and also through the development of cancer stem cells and drug resistance [52]. We found that apelin expression was not associated with the development and prognosis of RCC. Zhang et al. (2017) reported contradictory findings among two laboratory techniques with regards to apelin expression. In their first technique, there was no significant difference between RCC and adjacent normal tissue [27]. The second technique showed an upregulation of apelin expression in RCC compared to adjacent non-cancer tissue using the TCGA online database [27]. Recent studies, based on the TCGA and GTEx databases, have identified RCC patients with a high expression of apelin have a significant positive effect on OS [38]. Another study has also shown a very weak association of apelin with the tumour histological grade of RCC [37]. Moreover, autosomal dominant polycystic kidney disease (ADPKD) patients were characterised by lower apelin levels and higher copeptin levels when compared with healthy subjects (HS) [53]. Thus, apelin and copeptin showed a very good diagnostic profile in identifying ADPKD progression. Apelin is associated with kidney function decline in ADPKD, suggesting that it may be a new marker to predict kidney outcomes [53]. Contrary to the above findings, apelin was shown to be overexpressed in human colon adenomas and adenocarcinomas, endometrial and lung cancer, which are correlated with elevated cell proliferation, migration and invasion of cancerous cells [54]. These results suggest that apelin executes different functions in various malignancies through different signalling pathways and exhibits tissue-specific expression. In addition, patients with various cancer diagnoses had higher apelin levels compared with HS [55]. The increase in apelin was also associated with an increased risk of progression, and the increased risk was correlated with the presence of hyponatremia, a chronic kidney disease and obviously an advanced cancer stage [55].

### Strengths and Limitations

This systematic review is the first to provide cumulative current evidence regarding the role of visfatin, omentin-1, nesfatin-1 and apelin in RCC to the best of our knowledge. However, some limitations exist in this systematic review and meta-analysis. First, limited studies are included in the meta-analysis (six studies ) for two adipokines: nesfatin-1 and apelin. Specifically, small sample sizes and fewer than two studies were analysed for each protein at times. Secondly, full access to all data sets was not available, which is a limitation. Thirdly, significant heterogeneity was found in each analysis. This might be due to the sample collection and the method used for analysis. Even when the right meta-analytic techniques are applied and random effect models are used, heterogeneity could not be eliminated.

## 5. Conclusions

In conclusion, in this review, among the four adipokines studied, we observed that visfatin and nesfatin-1 can potentially act as a diagnostic biomarker for RCC. They can also be considered for use as a prognostic biomarker based on our review. This promising association needs further investigation, specifically in analysing in vitro and in vivo studies for visfatin and nesfatin-1, including their mechanism. Further prospective clinical studies would be required to confirm the possible value potential of visfatin, omentin-1, nesfatin-1 and apelin in RCC patients.

## Figures and Tables

**Table 1:** Characteristics of included studies in the systemic review and meta-analysis.

Reference	Author	Title	Country	Study Subject	Adipokine	Study Sample		Diagnostic Value/Prognostic Value (Stage/Grade)/Overall Survival (OS)/*p* Value	Studies Included in Meta-Analysis	Fixation Type	Antibody Used for Fixation	Scoring Procedure
						Serum/Plasma/Tissue/Cell Lines/Animal Model	Sample Size					
[27]	Zhang et al. (2017)	Association of leptin, visfatin, apelin, resistin and adiponectin with clear cell renal cell carcinoma	China	Human	Visfatin	Tissue	77 Controls 77 ccRCC	Control 25.24 ± 1.07 (77) Case 22.73 ± 0.88 (77) *p* < 0.01	No	N/A	N/A	N/A
[28]	Yamada Y et al. (2018)	Impact of novel oncogenic pathways regulated by antitumor miR-451a in renal cell carcinoma	Japan	Human	Visfatin	Tissue	261 RCC	*p* = 0.0138	No	N/A	N/A	N/A
[29]	Choi SH et al. (2016)	Identifying the emerging role of adipokine as a diagnostic and prognostic biomarker of renal cell carcinoma	Korea	Human	Visfatin	Plasma	25 Controls 54 ccRCC	Control 2.06 (1.52–2.68) (25) Case 2.26 (2.06–2.73) (54) *p* = 0.035	No	N/A	N/A	N/A
[30]	Shackelford RE et al. (2017)	Increased Nicotinamide Phosphoribosyltransferase and Cystathionine-β-Synthase in Renal Oncocytomas, Renal Urothelial Carcinoma, and Renal Clear Cell Carcinoma	USA	Human	Visfatin	Tissue microarrays (ccRCC Fuhrman grades: grade I–IV)	107 ccRCC	Not reported	No	Immunohistochemistry (IHC)	Mouse monoclonal antibody to human visfatin	The IHC score was the product of the percentage of cells stained multiplied by the intensity score, allowing for a maximal score of 9 and a minimal score of 0.
[31]	Shen X et al. (2016)	Circulating levels of adipocytokine omentin-1 in patients with renal cell cancer	China	Human	Omentin-1	Serum	42 Controls 41 RCC	Not reported	No	N/A	N/A	N/A
[32]	Fu H et al. (2017)	High NUCB2 expression level represents an independent negative prognostic factor in Chinese cohorts of non-metastatic clear cell renal cell carcinoma patients	China	Human	Nesfatin-1	non-metastasis (pT1–3N0M0) clear cell renal cell carcinoma (ccRCC)	434 ccRCC	Early stage (low expression = 198/335) 60.0% (high expression = 137/335) 40.0% Late stage (low expression = 47/99) 47.0% (high expression = 52/99) 53.0% *p* = 0.107 Early grade (low expression = 186/281) 66.0% (high expression = 95/281) 34.0% Late grade (low expression = 59/153) 39.0% (high expression = 94/153) 61.0% *p* ≤ 0.001 HR 3.464, 95% CI (1.859–6.454) *p* < 0.001	Yes	Immunohistochemistry (IHC)	Rabbit anti-NUCB2 polyclonal antibody	A semi-quantitative H-score was computed for each sample by multiplying the staining intensities (0: negative, 1: weak staining, 2: moderate staining, 3: strong staining) and distribution areas (0–100%), which ranged from 0 to 300.
[33]	Qi C et al. (2015)	Nucleobindin 2 expression is an independent prognostic factor for clear cell renal cell carcinoma	China	Human	Nesfatin-1	ccRCC tumours from patients	188 ccRCC	Early stage (low expression = 62/127) 48.8% (high expression = 65/127) 51.2% Late stage (low expression = 17/61) 27.9% (high expression = 44/61) 72.1% *p* = 0.006 Early grade (low expression = 63/134) 47.0% (high expression = 71/134) 53.0% Late grade (low expression = 16/54) 29.6% (high expression = 38/54) 70.4% *p* = 0.029 HR 4.545, 95% CI (2.122–9.733) *p* < 0.001	Yes	Immunohistochemistry (IHC)	Rabbit anti-NUCB2 polyclonal antibody	The score was the sum of the intensity and area scores, and on this basis staining was considered negative if the final score was 0–2 and positive if it was 3–4.
[34]	Xin R et al. (2022)	circ_001504 promotes the development of renal cell carcinoma by sponging microRNA-149 to increase NUCB2	China	Human	Nesfatin-1	Tissue	72 ccRCC	Not reported	No	N/A	N/A	N/A
[35]	Tao R et al. (2020)	Nucleobindin-2 enhances the epithelial-mesenchymal transition in renal cell carcinoma	China	In vitro, in vivo	Nesfatin-1	SK-RC-52 Renca cell BALB/c mice	n/a	Not reported	No	N/A	N/A	N/A
[36]	Xu H et al. (2018)	A novel function of NUCB2 in promoting the development and invasion of renal cell carcinoma	China	In vitro	Nesfatin-1	786-O ACHN HEK-293	n/a	Not reported	No	N/A	N/A	N/A
[37]	Tolkach Y et al. (2019)	Apelin and apelin receptor expression in renal cell carcinoma	Germany	Human	Apelin	Tissue	481 ccRCC	HR 1.6, 95% CI 1.2–2.2, *p* = 0.004	Yes	N/A	N/A	N/A
[38]	Bai S et al. (2020)	Construct a circRNA/miRNA/mRNA regulatory network to explore potential pathogenesis and therapy options of clear cell renal cell carcinoma	China	Human	Apelin	Tissue	258 ccRCC	HR 0.68, *p* = 0.012	Yes	N/A	N/A	N/A
[39]	Zhang N et al. (2020)	Identification of biomarkers of clear cell renal cell carcinoma by bioinformatics analysis	China	Human	Apelin	Tissue	72 Controls 525 ccRCC	Controls 9.64 ± 5.498 (72) transcript per million Cases 53.69 ± 27.36 (525) transcript per million	Yes	N/A	N/A	N/A
[27]	Zhang HP et al. (2017)	Association of leptin, visfatin, apelin, resistin and adiponectin with clear cell renal cell carcinoma	China	Human	Apelin	Tissue	77 Controls 77 ccRCC	Control 30.02 ± 2.02 (77) Case 28.31 ± 1.61 (77)	Yes	N/A	N/A	N/A

Abbreviations: ccRCC = clear cell renal cell carcinoma, RCC = renal cell carcinoma, NUCB2 = Nucleobindin 2.

**Table 2:** Summary of visfatin and omentin-1 studies.

Reference	Author	Country	Title	Sample Type	No of Participant	Outcome
Visfatin						
[28]	Yamada Y et al. (2018)	Japan	Impact of novel oncogenic pathways regulated by antitumor miR-451a in renal cell carcinoma	Tissue	261 RCC	High expression of visfatin was significantly associated with poor prognosis.
[29]	Choi SH et al. (2016)	Korea	Identifying the emerging role of adipokine as a diagnostic and prognostic biomarker of renal cell carcinoma	Plasma	54 ccRCC 25 Controls	Visfatin levels were higher in RCC patients (2.26 (2.06–2.73) than in normal healthy controls (2.06 (1.52–2.68), *p* = 0.035, and significantly associated with RCC severity (T stage). Expressed as the median (interquartile range).
[30]	Shackelford RE et al. (2017)	USA	Increased Nicotinamide Phosphoribosyltransferase and Cystathionine-β-Synthase in Renal Oncocytomas, Renal Urothelial Carcinoma, and Renal Clear Cell Carcinoma	Tissue	94 ccRCC	Visfatin protein levels increase in RCC at higher Fuhrman grades. RCC Fuhrman I (*n* = 44) 3.27 ± 0.13 RCC Fuhrman II (*n* = 28) 5.18 ± 0.32 RCC Fuhrman III (*n* = 13) 6.54 ± 0.54 RCC Fuhrman IV (*n* = 9) 8.67 ± 0.71 Expressed as mean ± standard error of measurement.
[27]	Zhang et al. (2017)	China	Association of leptin, visfatin, apelin, resistin and adiponectin with clear cell renal cell carcinoma	Tissue	77 Controls 77 ccRCC	Visfatin gene expression was upregulated in the ccRCC (22.73 ± 0.88) compared to adjacent normal tissue (25.24 ± 1.07), *p* < 0.01.
Omentin-1						
[31]	Shen X et al. (2016)	China	Circulating levels of adipocytokine omentin-1 in patients with renal cell cancer	Serum	41 RCC 42 Controls	The omentin-1 levels in healthy controls (9.86 ± 1.44) were higher compared to RCC patients (3.62 ± 0.76). Expressed in terms of ± standard error of measurement.

Abbreviations: ccRCC = clear cell renal cell carcinoma, RCC = renal cell carcinoma.

**Table 3:** Summary of Nesfatin-1 using in vivo and in vitro pre-clinical studies.

Reference	Author	Country	Title	No of Studies	Study Test	Outcome
In vivo						
[35]	Tao R et al. (2020)	China	Nucleobindin-2 enhances the epithelial-mesenchymal transition in renal cell carcinoma	10 control mice 10 knockdown mice	Transfection RT-PCR	Transfection with shRNA significantly decreased nesfatin-1 mRNA and protein levels and the growth of tumours in the nesfatin-1 KD group was significantly slower compared with the control group, indicating nesfatin-1 has a role in the growth of RCC.
In vitro						
[35]	Tao R et al. (2020)	China	Nucleobindin-2 enhances the epithelial-mesenchymal transition in renal cell carcinoma	-	Knockout RT-PCR Western blotting	Nesfatin-1 knockout in the RCC cell line inhibited cell migration, proliferation and invasion. Snail and Slug expression was significantly decreased in the nesfatin-1 knockout cells, suggesting that it may be involved in EMT through ZEB1 signalling.
[36]	Xu H et al. (2018)	China	A novel function of NUCB2 in promoting the development and invasion of renal cell carcinoma	-	Knockdown (KD) RT-PCR	Upon transfection, mRNA and protein levels of nesfatin-1 decreased significantly. The KD of nesfatin-1 induced an increased apoptotic rate in RCC. Nesfatin-1 KD cells also showed significantly reduced invasion.

Abbreviations: RCC = renal cell carcinoma, KD = knockdown, RT-PCR = Reverse transcription polymerase chain reaction, shRNA = Short Hairpin RNA, EMT = Epithelial–mesenchymal transition, mRNA = Messenger RNA.

## Data Availability

Not applicable.

## Supplementary Materials

The following supporting information can be downloaded at: https://www.mdpi.com/article/10.3390/diagnostics12123069/s1, Table S1: REMARK guideline; Table S2: ARRIVE guideline; Table S3: Quality assessment tools for in vitro studies adapted from Nature.
