# Supplementary material for: Potential Value of Visfatin, Omentin-1, Nesfatin-1 and Apelin in Renal Cell Carcinoma (RCC): A Systematic Review and Meta-Analysis
Source: Diagnostics (Basel). 2022 Dec 6;12(12):3069. doi: 10.3390/diagnostics12123069 (PMC9776786; doi:10.3390/diagnostics12123069)
Supplement: Supplementary file 1 [file diagnostics-12-03069-s001.zip › diagnostics-1963647-supplementary.pdf]

Systematic review

Visfatin, Omentin-1, Nesfatin-1 and Apelin Potential Value in Renal Cell Carcinoma (RCC): A Systematic Review and Meta-Analysis

Sugania Malar Chinapayan [1], Shanggar Kuppusamy [1], Ning Yi Yap [2], Komathi Perumal [3], Glenda Gobe [4,5,6] and Retnagow Rajandram [1,*]

[1] Department of Surgery, Faculty of Medicine, Universiti Malaya, Kuala Lumpur, Malaysia
[2] Laboratory, Subang Jaya Medical Centre, Selangor, Malaysia
[3] Epink health Sdn Bhd, Shah Alam, Selangor
[4] NHMRC CKD CRE (CKD.QLD), The University of Queensland, Brisbane 4067, Australia
[5] Faculty of Medicine, The University of Queensland, Brisbane 4067, Australia
[6] Kidney Disease Research Collaborative, Translational Research Institute, Princess Alexandra Hospital, The University of Queensland, Brisbane 4102, Australia
* Correspondence: retnagowri@ummc.edu.my

Supplementary Materials

Table S1. REMARK guideline.

	Marker description	Patient characteristics	Information about treatment	Biological material & preservation method	Assay method	Sample selection & follow-up time	Clinical endpoint definition	Variables included in analysis	Rational for sample size	Specification of statistical methods	Handling of marker values	Description of flow of patients through study	Basic demographic characteristics	Relation marker to standard variables	Univariable analysis	Multivariable analysis	Marker & standard variables	Further investigations	Interpretations of results & limitations of study	Implications for further research	Score

REMARK item 1 2 3 4 5 6 7 8 9 10 11 12 13 14 15 16 17 18 19 20 SCORE

REFERENCE

TITLE

Nucleobindin-2 enhances the epithelial-mesenchymal transition in renal cell carcinoma	Tao R et al, 2020	0.5	1	0	1	1	0	1	0.5	1	1	1	0	0	1	0	0	1	0.5	0.5	0.5	11.5/20
High NUCB2 expression level represents an independent negative prognostic factor in Chinese cohorts of non-metastatic clear cell renal cell carcinoma patients	Fu H et al, 2017	1	1	0	0	1	1	1	0	1	1	1	1	1	1	1	1	0.5	O	1	0.5	18/20
Nucleobindin 2 expression is an independent prognostic factor for clear cell renal cell carcinoma	Qi C et al,2015	1	0.5	0	0	1	0.5	1	0	1	1	0.5	0.5	1	1	1	1	0.5	0.5	1	0.5	13.5/20
Apelin and apelin receptor expression in renal cell carcinoma	Tolkach Y et al, 2019	1	0	0	0.5	1	0.5	0.5	0	1	0.5	1	1	1	1	1	0.5	1	0.5	1	0.5	1.35/20
Association of leptin, visfatin, apelin, resistin and adiponectin with clear cell renal cell carcinoma	Zhang HP et al, 2017	1	1	0	1	1	0	0	0	1	1	1	0	0	0	0	0	1	0	1	1	10/20
Increased Nicotinamide Phosphoribosyltransferase and Cystathionine-β-Synthase in Renal Oncocytomas, Renal Urothelial Carcinoma, and Renal Clear Cell Carcinoma	Shackelford RE et al, 2017	0	1	0	0	1	0	1	0	1	1	1	1	1	1	0	0	0	0	0.5	1	10.5/20

Identifying the emerging role of adipokine as a diagnostic and prognostic biomarker of renal cell carcinoma	Choi SH et al, 2016	0.5	1	0	1	0	0.5	0	0	1	1	1	1	1	1	0	0	0.5	0.5	1	1	12/20
circ_001504 promotes the development of renal cell carcinoma by sponging microRNA-149 to quaincrease NUCB2	Rui Xin et al,2020	1	0	1	1	1	0	0	0	1	1	1	0	0	0	1	0	0	0	1	1	10/20
Identification of biomarkers of clear cell renal cell carcinoma by bioinformatics analysis	Zhang N et al, 2020	0	0	0	0	1	0	1	1	1	1	1	0.5	1	0	1	0	0	0	1	1	11/20
Construct a circRNA/miRNA/mRNA regulatory network to explore potential pathogenesis and therapy options of clear cell renal cell carcinoma	Shuheng Bai et al, 2020	1	0	0	0	0	0	1	1	1	1	1	0	0	0	1	0	1	1	1	1	11/20
Impact of novel oncogenic pathways regulated by antitumor miR-451a in renal cell carcinoma	Yamada Y et al,2018	1	1	0	0	1	0	0	1	1	1	1	0	1	1	1	0	0	0	1	0	11/20
Circulating levels of adipocytokine omentin-1 in patients with renal cell cancer	Shen et al., 2016	1	1	0	1	1	0	1	1	1	1	1	0	0.5	1	0	0	0	0	1	1	12.5

Table S2. ARRIVE guideline

TITLE	REFERENCE	title	abstract	Into-background	Intro-objective	Method-ethical statement	Method-study design	Method-experimental procedure	Method-experimental animal analysis	Method-housing and husbandry	Method-sample size	Method-allocating animal to experimental group	Method-experimental outcome	Method-statistical method characteristics	Result-base line data	Results-numbers analysed	Result-outcome and estimation	Result-adverse event	interpretation/scientific implication	Discussion-generalization	Discussion-funding	Score
ARRIVE item		1	2	3	4	5	6	7	8	9	10	11	12	13	14	15	16	17	18	19	20	SCORE
Nucleobindin-2 enhances the epithelial-mesenchymal transition in renal cell carcinoma	Tao R et al, 2020	1	1	1	1	2	1	2	2	2	1	1	2	1	1	0	1	0	0	1	2	17/20

Table S3. Quality assessment tools for in-vitro studies adapted items from Nature.

		Sample Selection & Experimental Setup					Reagents & Cells					Sample Size		Allocation to groups	Allocation Concealment-experiment	Blinded Assessment of Outcome	Attrition	
TITLE	REFERENCE	sample collection	experimental unit	Experimental replicates	sex of the cells	source of cell lines	authentication	tested for mycoplasma contamination	Tyoes of cell lines	passage number	Profiled antibody	Discription of sample size	Sample size calculation	methods of allocation	investigators blinded to the group allocation	investigators blinded to the group allocation	Sample excluded	exclusion criteria pre-defined
Nucleobindin-2 enhances the epithelial-mesenchymal transition in renal cell carcinoma	Tao R et al, 2020	Y	Y	Y	Y	Y	N/A	N/A	Y	N	Y	Y	N	UN	Y	Y	N	N
A novel function of NUCB2 in promoting the development and	Xu H et al, 2018	Y	Y	Y	N	Y	N/A	N/A	Y	N	UC	Y	N	Y	Y	Y	N	N

invasion of renal cell carcinoma
